# A CARE-compliant article: Unicentric Castleman disease presenting as a retroperitoneal mass of the upper edge of the pancreas: A case report

**DOI:** 10.1097/MD.0000000000019515

**Published:** 2020-03-13

**Authors:** You-Xin Zhou, Yong Ji, Sheng Wu

**Affiliations:** a Department of General Surgery, People Hospital of Jingjiang; b Department of Pathology, People Hospital of Jingjiang, YangZhou University Medical Academy, Jingjiang, Taizhou, China.

**Keywords:** case report, peripancreatic, retroperitoneal, unicentric Castleman disease

## Abstract

**Rationale:**

Castleman disease (CD) is a rare lymphoproliferative disease with a poorly understood etiology. The occurrence of CD in the abdominal cavity is very rare, especially in the retroperitoneal peripancreatic region.

**Patient concerns:**

A 33-year-old woman was referred to our department on March 1, 2018 for a detailed physical examination due to retroperitoneal peripancreatic lymph node enlargement over 15 days.

**Diagnosis:**

Enhanced magnetic resonance imaging of the epigastrium showed the mass with abundant blood supply is located between the liver and the stomach in the upper margin of the pancreas. Postoperative pathological examination revealed CD, type of unicentric Castleman disease.

**Interventions:**

We performed an open surgery on this patient and completely removed the mass. There was no postoperative radiochemotherapy.

**Outcomes:**

The patient was followed-up for more than 12 months after the operation and showed good recovery.

**Lessons:**

CD is a rare disorder that is hard to diagnose early and complete resection of the tumor is still the most effective treatment.

## 1 Introduction

Castleman disease (CD) is a rare nonclonal lymph proliferative disorder of unknown etiology, which was first described as a pathologic entity in 1954 and later defined by Dr Benjamin Castleman in 1956.^[1,5]^ CD is a rare lymphoproliferative disorder that can involve single (unicentric) or multiple lymph nodes (multicentric). In addition, it can be classified into 3 histopathological patterns: hyaline-vascular (HV) type, plasma cell (PC) type, and mixed variant. Type of HV is the most common. CD, which develops in all lymph nodes, especially in the mediastinum, can also occur in the cervical, retroperitoneal, and axillary regions. However, invasion into the retroperitoneal peripancreatic region is quite rare.^[6,8]^ We herein report a rare unicentric Castleman disease (UCD) located in the superior margin of the pancreas.

## 2 Case report

A 33-year-old woman was referred to our department on March 1, 2018 for a detailed physical examination due to retroperitoneal peripancreatic lymph node enlargement over 15 days and she had no apparent discomfort. The patient had no significant medical history and had no significant family history. The physical examination showed no positive signs.

**Figure 1:**
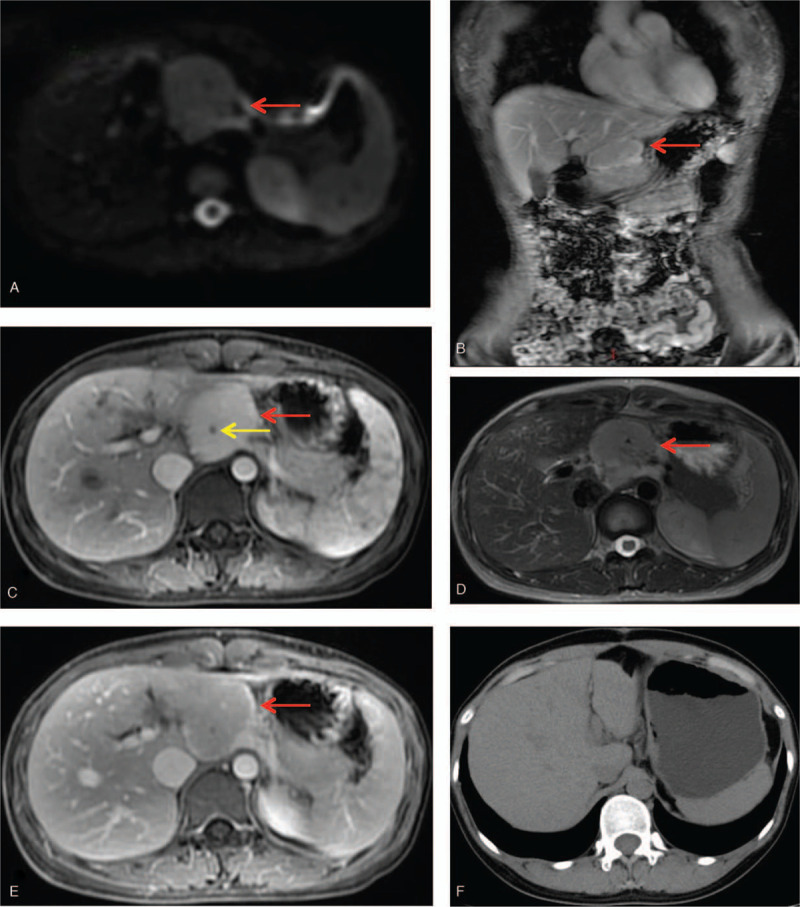
Magnetic resonance imaging of the lesion (red arrow) in Case; Microcalcifications (yellow arrow). (A) Diffusion weighted imaging; (B) coronal scan; (C) Transverse T1-weighted fat-suppression sequence (arterial phase); (D) Transverse T2-weighted fat-suppression sequence; (E) Transverse T1-weighted fat-suppression sequence (Venous phase); (F) CT images reviewed 1 yr later and the mass disappeared without recurrence.

Laboratory examinations revealed that the patient was negative for the human immunodeficiency virus (HIV) antibody. Karyotype analysis of bone marrow chromosomes revealed 10 normal metaphases and the results of bone marrow immunotyping (CD series) – lymphoma (type to be determined) showed no abnormal phenotypes in NK and T lymphocytes, and no clonal abnormalities in PCs and B lymphocytes. Classification of bone marrow cells, tumor markers, and other laboratory test results were also within the normal range. Enhanced magnetic resonance imaging of the epigastrium showed the mass with abundant blood supply is located between the left lobe of liver and the stomach in the upper margin of the pancreas (Fig. 1A–E).

The patient underwent laparotomy under general anesthesia. When the gastrocolic ligament was incised, a tumor with abundant blood supply was discovered closely adhered to the upper edge of the pancreas. The tumor volume was about 5.0 cm × 4.0 cm × 4.0 cm, had a soft flexible texture and a clear boundary. The mass was completely removed along the mass and surrounding tissue gap. The patient did not receive postoperative adjuvant radiotherapy and chemotherapy.

**Figure 2:**
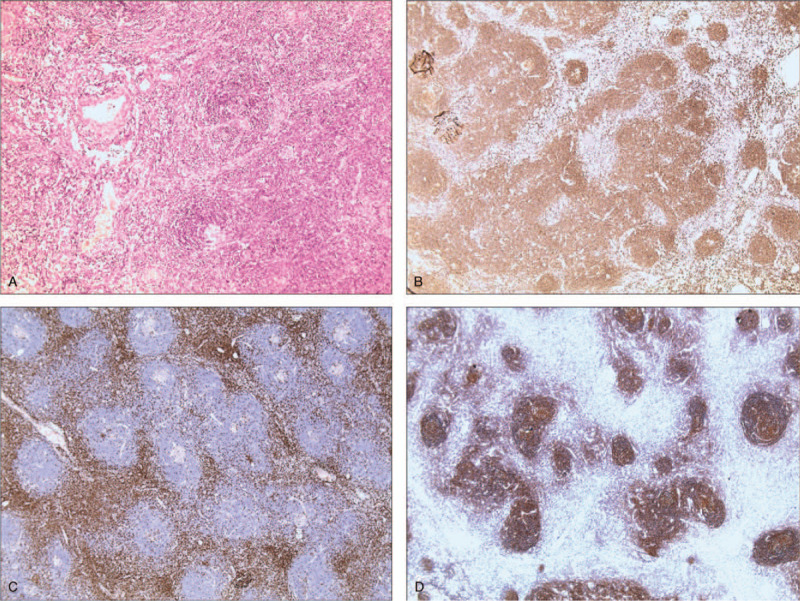
Histological examination. (A) (HE, ×100) Germinal center degeneration; Proliferated blood vessels inserted into follicular germinal center; Sclerotic vessels; Sign of concentric rings; (B) CD20(+); (C) CD3(+); (D) CD21(+). HE = hematoxylin and eosin.

Immunohistochemical results: follicular dendritic cells CD21(+), CD23(+); interfollicular zone cells CD3(+), CD43(+), ki67(+)10%; follicular germinal center cells CD20(+), CD79ɑ(+), ki67(+)70%, blc-2(−), CD10(+), bcl-6(+) (Fig. 2). Postoperative pathology indicated that the retroperitoneal peripancreatic mass was compatible with CD (Type of HV).

The patient recovered well after the operation. The patient underwent abdominal computed tomography (CT) reexamination on March 4, 2019 (Fig. 1F). The mass previously located between the left lobe of the liver and the stomach disappeared without recurrence.

## 3 Discussion

CD is a rare lymphoproliferative disease with a favorable prognosis; however, the pathogenesis of this disease is unclear. Munshi et al^[11]^ showed that the incidence of CD is approximately 21/million. The disease can occur in any age group, but it is more common in patients aged 30 to 50, with an average age of 43,^[9]^ without significant gender differences.^[19]^ The distribution of CD was reported as 60% in the thorax, 14% in the cervical area, 11% in the abdomen, and 4% in the axillary region^[2]^; however, its occurrence in the retroperitoneal peripancreatic region is more rare.^[8,13]^ The etiology and pathogenesis of CD is largely unexplored. The etiology of CD is thought to be related to herpes simplex virus 8 and HIV.^[13,15]^ Another report confirmed that the pathogenesis of CD was closely related to the proliferation of T cells and B cells stimulated by interleukin 6.^[12]^

The majority of patients with UCD present with isolated lymphadenopathy and may; therefore, be asymptomatic or have symptoms relating to mass effects on surrounding structures. From a systematic case review including 278 patients with UCD, the mean size of involved lymph nodes at baseline was 5.5 cm, compared to 3.8 cm for multicentric Castleman disease (MCD) cases.^[19]^ Systemic symptoms are a common feature of MCD and MCD mainly manifests as a painless enlargement of deep or superficial lymph nodes, and may be accompanied by fever, fatigue, anemia, night sweats, and an accelerated erythrocyte sedimentation rate.^[18]^ Our patient had no any complaints of discomfort.

Because the tumors are located behind the peritoneum, we cannot perform preoperative biopsy. So, retroperitoneal peripancreatic UCD is usually concealed and difficult to diagnose. The radiological appearance of CD is nonspecific. Imaging manifestations of the disease are very difficult to distinguish from other diseases, including neuroendocrine tumor, paraganglioma, or lymphoma.^[17]^ However, CT, MRI, and ultrasound can still provide reference values for the diagnosis of retroperitoneal UCD. On CT imaging, retroperitoneal UCD is generally a well-defined mass with different morphologies, such as elliptical, kidney-shaped, and dumbbell-shaped, which can easily be distinguished from a spherical paraganglioma. The plain CT scan images of this disease show low or equal density lesions. The majority of lesions exhibit inhomogeneous enhancement on enhanced CT images. In addition, some tumors have a rich vascular supply, accompanied by Microcalcifications.^[14]^ On the magnetic resonance imaging (MRI), most retroperitoneal UCD lesions show an isointense or hypo-intense signal on T1WI, and a slight hyperintense or hyperintense signal on T2WI. In addition, ultrasound also has some advantages in estimating the location of the mass and its adjacent feeding vessels^[8]^ and a homogeneous hypoechogenic formation is commonly shown on ultrasound.^[4]^

Histological evaluation plays a vital role in the exact diagnosis of CD. The histopathological features consistent with CD diagnosis include abnormal regressed or hyperplastic germinal centers, follicular dendritic cells prominence, hypervascularisation, expanded mantle zones, and interfollicular plasmacytosis. Two major pathological features can be defined: a HV subtype and a plasmacytic (PC) subtype, with some lesions exhibiting mixed characteristics.^[3]^ The percentage of HV type and PC type is approximately 90% and 10%, respectively. Mixed type cases are rarely reported. Most HV type cases manifest as UCD, while PC type cases usually occur in the form of multicentric CD.^[10]^ The characteristics of the hyaline vascular type include considerable enlarged lymphoid follicular proliferation at various levels of maturity. In addition, lymphoid follicles are normally scattered throughout the tissue. The PC type is almost related to the multicentric form of CD. This type shows less vascularity and is characterized by sheets of mature PCs within the interfollicular tissues surrounding larger germinal centers.^[7]^ Histological examination of our case revealed enlarged lymph nodes consisting of multiple follicles of different sizes, degeneration of germinal centers and hyaline degeneration of blood vessels. High power microscopy showed that the follicles were surrounded by concentric rings of lymphocytes.

Surgery remains the gold standard if the lesion is amenable to complete resection.^[20]^ Complete resection provides confirmation of the diagnosis with histological evaluation. If complete surgery resection is not possible, debulking of the main lesion can be considered to reduce local symptoms. Residual associated small lymph nodes may involute after surgery.^[21]^ Radiotherapy and chemotherapy can be used for unresectable or recurrent tumors. But their efficacy still needs to be confirmed by further studies. At present, there is no standard protocol for predicting the prognosis and effectively managing UCD. In asymptomatic patients for whom surgery is impossible or too mutilating, watch-and-wait can be considered.^[16]^ Our patient underwent complete resection of the tumor and did not undergo radiochemotherapy after surgery. In this report, we described 1 rare case of retroperitoneal peripancreatic UCD, which will contribute to an improvement in the understanding of UCD and provide a significant reference for future cases.

## Acknowledgments

In this study, no special individuals and organizations need to be appreciated.

## Author contributions

**Resources:** Sheng Wu.

**Supervision:** Yong Ji, You-Xin Zhou.

**Writing – original draft:** You-Xin Zhou.

**Writing – review and editing:** You-Xin Zhou.

You-Xin Zhou orcid: 0000-0001-8498-709X.
